# Finite element analysis of shape and thickness variations in patient-specific implants for t-shaped genioplasty

**DOI:** 10.4317/medoral.27065

**Published:** 2025-03-23

**Authors:** Emine Fulya Akkoyun, Taha Pergel

**Affiliations:** 1 DDS, PhD. Assistant Professor. Department of Oral and Maxillofacial Surgery, Faculty of Dentistry, Bezmialem Vakif University, Istanbul, Turkiye; 2 DDS. Assistant Professor. Department of Oral and Maxillofacial Surgery, Faculty of Dentistry, Bezmialem Vakif University, Istanbul, Turkiye

## Abstract

**Background:**

T-shaped genioplasty is a versatile surgical technique used to correct chin deformities by altering its vertical, transverse, and sagittal dimensions. Despite numerous advancements in patient-specific implants (PSIs), the biomechanical impact of PSI thickness and the number of screws used for fixation remain unexplored. This study aims to evaluate the effects of PSI thickness and screw conFigurations on fixation stability in T-shaped genioplasty using finite element analysis.

**Material and Methods:**

Mandibular computed tomography data were used to construct 12 three-dimensional models with varying PSI thicknesses (0.6 mm, 0.9 mm, and 1.2 mm) and screw conFigurations (five, six, seven, and eight screws). T-shaped osteotomies were applied to create narrowing and widening genioplasty models, with iliac bone grafts placed in widening scenarios. Horizontal forces of 50 N were applied bilaterally, and fixation stability was evaluated using von Mises stress and bone displacement. Fixation was considered sTable when bone displacement amounts were below 1 mm.

**Results:**

In narrowing models, fixation stability was achieved with five screws and a 1.2 mm PSI or seven screws with a 0.9 mm or 1.2 mm PSI, showing stress values within titanium's yield strength limits. For widening models, sTable fixation was achieved with six screws and a 0.9 mm or 1.2 mm PSI, or eight screws across all tested thicknesses. Bone displacement was minimal with thicker PSIs and higher screw counts, demonstrating improved stability.

**Conclusions:**

This study highlights the importance of PSI customization in optimizing fixation stability in T-shaped genioplasty. A minimum of five screws with a 1.2 mm plate or seven screws with a 0.9 mm plate is suggested for narrowing, while six screws with a 0.9 mm plate or eight screws with any tested thickness are sufficient for widening. Future research should address combined movements, dynamic loading, and long-term outcomes to refine PSI fixation strategies further.

** Key words:**Finite element analysis, genioplasty, patient specific modeling, bone plates.

## Introduction

Genioplasty, an orthognathic surgical procedure applied to correct chin deformities, is based on altering the vertical, transverse, and sagittal dimensions of the chin’s shape (1). The chin is one of the most significant structures contributing to the overall facial aesthetics and harmony, as well as to the appeal of the facial profile (2). The anatomy is highly variable, and while its features can sometimes be a distinguishing characteristic of a person, in other cases, it may be a pathological element requiring surgical treatment.

Since intraoral genioplasty was introduced by Trauner and Obwegeser (3) in 1957, numerous surgical techniques have been developed to address various chin types. In 1964, Converse and Wood-Smith (4) presented several approaches to horizontal osteotomy. In 1990, a T-shaped genioplasty method was introduced, enabling vertical elongation or shortening and horizontal widening or narrowing of the chin (5). Recently, the most used technique is sliding genioplasty. Other techniques include genioplasties for chin setback, reduction, vertical lengthening, narrowing, or widening (6). In 2023, Ramieri *et al*. reported a technique named “Tetris genioplasty” (7). In deciding the appropriate technique for each patient, the surgeon assesses the specific deformity and the desired outcome.

The wire osteosynthesis technique was initially used for the stabilization of the genial segment (8). However, due to undesirable outcomes such as insufficient rigidity and a high incidence of relapses with wire fixation, recently more reliable methods were adopted. Rigid fixation techniques, which include metal plates, screw systems, and various combinations thereof, have been in use for many years with proven safety (9). In osteotomies performed without dissecting the muscle attachments on the lingual side of the distal bone segment, the connected muscles exert a traction force. These muscles include the genioglossus, mylohyoid, anterior belly of the digastric, and geniohyoid muscles (10). As a result, these muscles can influence the stability of the fixation. In oral and maxillofacial surgery, screw-plate systems are used for the treatment of mandibular fractures and orthognathic surgery. Despite their long-standing use and establishment as a standard treatment, these systems can fail due to factors such as excessive loading, inadequate plate adaptation and fixation to the bone during surgery, material design, fabrication, and the purity of the plate material (11). During the surgery, the heavy bending forces required to adapt the plate to bone morphology can cause fixation fatigue in the material (12) leading to breakage of the fixation material. This compromises stability, often necessitating the removal and replacement of the osteosynthesis material.

Advances in reconstructive surgical techniques for the head and neck region have allowed for a surgical approach that yields significant improvements in aesthetic and functional reconstruction, resulting in fewer comorbidities and better recovery for patients (13). Less aggressive surgical techniques, additive manufacturing, and digital workflows have significantly enhanced comprehensive reconstruction of the facial skeleton, including the surgical treatment of the jaws (13,14). Mazzoni *et al*. developed a patient-specific, fully guided computer-assisted orthognathic surgery planning project, proposing virtual surgical planning (VSP) and patient-specific implants (PSI) as promising methods for future orthognathic surgery (15). It has been demonstrated that with improved reliability of VSP, especially in patients with asymmetrical deformities, PSIs can accurately achieve the planned three-dimensional (3D) position during jaw surgeries (16). In this context, VSP, along with computer-aided design and manufacturing (CAD-CAM) technology, cutting guides, and patient-specific titanium mini plates, has introduced a new dimension to the surgical treatment of maxillomandibular deformities. Numerous studies in literature affirm the accuracy of PSIs in achieving precise clinical and surgical outcomes (17,18).

Although numerous studies (2,8,11,14,17,18) have evaluated genioplasty, none have specifically investigated the impact of screw-plate systems on stability following T-shaped genioplasty. Furthermore, the optimal thickness and the impact of screw number on stability of PSIs has not been explored. This study aims to evaluate the effects of variations in PSI thickness and number of screw holes on PSI deformation and stability in T-shaped genioplasty.

## Material and Methods

- Software and Hardware Specifications

A mandibular computed tomography (CT) image from the database of the Bezmialem Vakif University Faculty of Dentistry was used to evaluate PSIs in T-shaped genioplasty through FEA. The arrangement of the 3D mesh structure and its conversion into a mathematically appropriate solid mesh structure, the creation of 3D FEA models, and the finite element stress analysis procedures were carried out on HP workstations equipped with an INTEL Xeon E-2286 processor with a 2.40 GHz clock speed and 64 GB ECC memory.

- Modeling of Cortical and Trabecular Bone

The CT data of an adult patient from the database was reconstructed with a slice thickness of 0.1 mm. The reconstructed CT data was transferred in DICOM (Digital Imaging and Communications in Medicine) format to the 3DSlicer software. In 3DSlicer, the DICOM CT data was segmented based on appropriate Hounsfield values and converted into 3D models. The models were then exported in STL (Standard Tessellation Language) format. The 3D models were imported into ANSYS Spaceclaim, where a 2 mm offset was applied to create a 2 mm thick cortical bone around the mandibular bone model. Using the inner surface of the cortical bone as a reference, the trabecular bone was generated. All prepared models were placed in the correct coordinates within the 3D space in ANSYS Spaceclaim, completing the modeling process.

- Modeling of Screws, PSIs, and Grafts, and Preparation of Simulation Models

The 3D CAD models of the screws (2.0 mm in diameter and 7 mm in length), PSIs, and graft used in the study were created in ANSYS Spaceclaim software. To enable force transmission between the models, alignment of the mesh structures was performed in ANSYS Workbench software.

- Simulation Models

**Figure 1:**
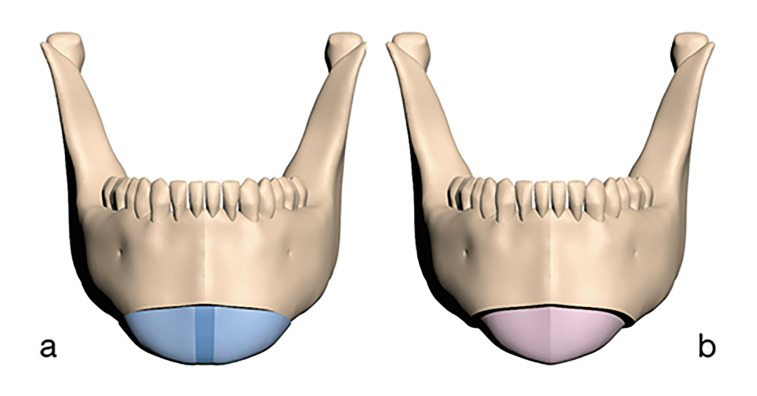
Widening (a) and narrowing (b) genioplasty models.

A T-shaped osteotomy with a 0.6 mm osteotomy line was applied to the cortical and trabecular bone in the chin area to create PSI models for widening and narrowing genioplasty. For the widening procedure, iliac bone graft models were created to fill a 4 mm gap established between the segments. For the narrowing procedure, a 4 mm-wide bone section was removed from the center of the chin after osteotomy, and the remaining chin bone segments were repositioned and assembled (Fig. 1).

**Figure 2:**
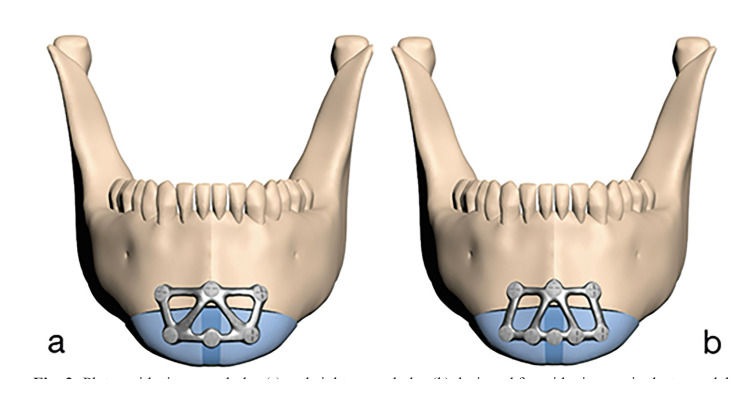
Plates with six screw holes (a) and eight screw holes (b) designed for widening genioplasty models.

**Figure 3:**
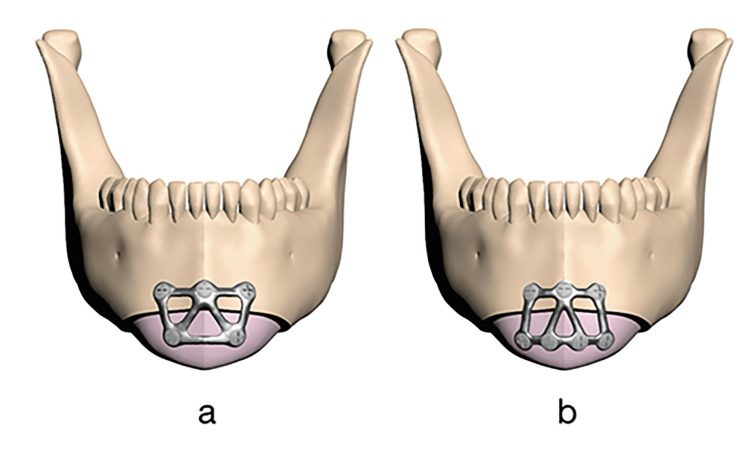
Plates with five screw holes (a) and seven screw holes (b) designed for narrowing genioplasty models.

The holes in the PSIs were positioned horizontally to center the segments, with six and eight screws for widening genioplasty on the distal chin segments and iliac bone (Fig. 2), and five and seven screws for narrowing genioplasty (Fig. 3). Three screw holes were positioned on the upper bone segment in all models. All screws were positioned 4 mm from the osteotomy line. PSIs were modeled according to parameters of 0.6 mm, 0.9 mm, and 1.2 mm thickness and 2 mm width.

- Formulation of the Mathematical Model

After the modeling process was completed in ANSYS Spaceclaim software, the models were mathematically generated and prepared for analysis in ANSYS Workbench software. To conduct the analyses, the mathematical models prepared in ANSYS Workbench were transferred to the LS-DYNA solver.

- Material Properties

In the analysis, linear material properties, characterized by the elastic modulus and Poisson's ratio of the given materials, were used (Table 1) (11,19). The material properties of the analyzed model were defined numerically. For subsequent analyses, the yield strength of titanium has been accepted as 830-900 MPa (11).

- Boundary and Loading Conditions

In all models, a total horizontal force of 100 N was applied to the chin area, distributed across the entire surface, with 50 N directed lingually from both the right and left sides of the midline. The forces were distributed across surrounding nodal points to prevent stress singularity in the loading regions. The models were fixed by constraining all degrees of freedom at the nodal points in the mandibular condyle area, restricting movement in all three axes. Under these specified force and boundary conditions, 12 linear static analyses were conducted.

- Quantitative Model Data

Information for the twelve analysis models created is provided in Table 2.

- Assembly of Systems and Interconnection of Components

In all analyses, a bonded contact type was defined between contacting components. This approach assumes that the parts move with full correlation during motion.

- Analysis

After force application, FEA in ANSYS software was used to investigate the stress values on fixation systems. The von Mises stress of PSIs and screws and bone displacement amount in the segments were calculated. Fixation was considered sTable when bone displacement at the osteotomy line between bone fragments was below 1 mm.

## Results

The von Mises stress values on the PSIs with various thickness and number of screws, as well as on their respective screws, after the application of horizontal loads were shown in Table 3. It was observed that as PSI thickness and number of screws increased, the amount of von Mises stress on both PSIs and screws decreased. The lowest amounts of bone displacement were observed in the model with a thickness of 1.2 mm and with seven screws for narrowing and eight for widening (0.92 mm and 0.79 mm, respectively). In the narrowing models, fixation stability was achieved with conFigurations that included either five screws and a 1.2 mm thickness PSI or seven screws with a PSI thickness of either 0.9 mm or 1.2 mm. These conFigurations demonstrated von Mises stress values (474.905 MPa, 805.946 MPa, and 420.219 MPa, respectively) within the yield strength range of titanium (830-900 MPa) and bone displacement amounts below 1 mm. In the widening models, the conFigurations with six screws and either 0.9 mm or 1.2 mm thickness, as well as eight screws across all tested thicknesses (0.6 mm, 0.9 mm, and 1.2 mm), provided sTable fixation with stress values remaining within the titanium yield strength limits (824.526 MPa, 437.387 MPa, and 241.779 MPa, respectively) and bone displacement amounts below 1 mm.

## Discussion

FEA was conducted on 12 models, each featuring PSIs of varying thicknesses and the number of screws specifically for genioplasty. Force applications were performed, and Table 3 presents the models with the highest von Mises stress values in the PSIs and screws. In the narrowing models, fixation stability was achieved using conFigurations with either five screws and a 1.2 mm thick PSI or seven screws with PSI thicknesses of 0.9 mm or 1.2 mm. These setups exhibited von Mises stress values within titanium’s yield strength limits, ensuring adequate resistance to deformation. For the widening models, conFigurations with six screws and PSI thicknesses of either 0.9 mm or 1.2 mm, as well as eight screws across all tested thicknesses, provided sTable fixation with stress levels remaining within titanium’s yield strength limits. Consequently, for narrowing applications, a conFiguration with five screws and a 1.2 mm PSI or seven screws with at least a 0.9 mm PSI could be suggested for achieving optimal fixation. For widening applications, six screws with a 0.9 mm thickness or eight screws with any thickness from 0.6 mm to 1.2 mm might be considered sufficient to ensure secure fixation. Other designs may be less suiTable for clinical use due to the potential risks of PSI deformation, screw loosening, and bone displacement.

In recent years, there has been an increase in demand for corrective surgeries to address facial asymmetries, in which the chin plays a significant role (20). Chin asymmetry presents a considerable challenge for the maxillofacial surgeon, who must explore the most effective techniques to ensure an optimal aesthetic outcome (21). In 1990, Grime *et al*. described the T-shaped genioplasty method (5). Jegal *et al*. reported using this technique to achieve vertical elongation or shortening, as well as horizontal widening or narrowing, of the chin (22). With this approach, 3D correction of chin deformities was possible in a single surgical procedure, and researchers achieved satisfactory results. The T-shaped genioplasty applied by the authors is a flexible method, adapTable to patients’ needs and suiTable for anteroposterior, lateral, and superoinferior corrections based on 3D modifications. According to the authors, the T-shaped osteotomy was the most beneficial approach, given its flexibility in correction and its alignment with the preoperative analysis of the chin deformity. Currently, no studies have evaluated the biomechanical effects of PSIs, plate thickness, or number of screw holes on the plate and bone segments in T-shaped genioplasty. Therefore, this study was designed to address this gap.

This customization allows for an ideal design and optimization of the number of screws used, reducing the need for long plates with numerous screw holes that are prone to stress concentration and potential breakage in advancement surgeries. In contrast, PSIs often require fewer screws to achieve sufficient fixation and stabilization of the mobile bone segment, enhancing plate resistance (11). Although the risk of breakage in bent standard plates is generally low, it can increase in patients with parafunctional habits, where chewing forces may reach two to three times the usual load. Additionally, titanium quality influences the risk of permanent deformation. While irreversible plastic deformation may not always lead to plate breakage, its occurrence during repetitive chewing cycles can impair bone healing by increasing mobility between mandibular segments (11). The suprahyoid muscles’ continuous activation on the bone segment further risks altering the segment's position during healing after chin advancement. Therefore, better plate adaptation to the segment reduces the risk of undesired displacement. Ramos *et al*. demonstrated that a higher number of screws improves stress distribution (11). Comparing two pre-bent plate systems, they reported that systems with more screws exhibited lower stress levels due to improved adaptation. However, another study suggested that excessive screws could weaken the bone segment (23). Given these findings, this study aims to provide insights into the ideal number of screws for stabilization in PSIs and their biomechanical impact on both the plate and bone segments, addressing a crucial gap in current literature.

Visibly prominent differences exist between the appearance of a typically male or female chin (24). The male chin is generally broader and wider, has greater anterior projection than the female chin and is about 17% taller - traits that collectively contribute to a larger and more square appearance. The female chin, on the other hand, is typically narrower, pointed, tapered, and shorter (25). Because of its importance in gender identification, chin contouring is a key aspect of facial feminization surgery. Adjustments such as lowering the height of the chin, reducing its width, or altering its anterior projection by even a few millimeters can dramatically influence the perception of femininity, particularly in transgender patients (6).

Narrowing genioplasty is performed more frequently than widening genioplasty and creates a more defined, tapered chin appearance (26). A prominent mandible and a square facial contour in women are often perceived as masculine and less attractive by many individuals. Narrowing genioplasty, performed alone or alongside mandible reduction, creates a slimmer lower facial appearance and enhances a more feminine contour (6). To narrow the chin, a central resection is performed, with the amount of bone removal tailored to the patient's specific needs. Park and Noh reported in their clinical applications that this amount varies between 6 and 12 mm (6). In cases where widening is required, a straight midline osteotomy is performed to separate the genium in two pieces with placement of a bone graft in between (26). Distraction osteogenesis for widening was also reported (27). In this study, narrowing genioplasty models involved a central bone resection of 4 mm, based on compatibility with available CT data. Similarly, for the widening procedure, a 4 mm gap was created and filled with an iliac bone block graft. Finite element analyses (FEM) were conducted to evaluate the biomechanical effects of these scenarios. These findings provide a critical foundation for optimizing patient-specific implant designs and improving clinical outcomes in T-shaped genioplasty. Future studies could expand on this work by exploring additional factors such as dynamic loading, bone density, and long-term outcomes.

This study focused solely on chin widening and narrowing procedures, without evaluating fixation stability in cases where these movements are combined with advancements, which are often required in clinical practice. As such, further studies are needed to investigate fixation stability in scenarios involving combined movements. Additionally, this study did not include a control group for comparison, which limits the ability to fully contextualize the findings. Despite these limitations, the study provides valuable insights into how bone segment stabilization can be achieved following widening or narrowing procedures, offering a foundation for future research.

## Conclusions

This study provides a detailed biomechanical analysis of patient-specific implants (PSIs) in T-shaped genioplasty, focusing on the effects of shape, thickness, and screw conFigurations on fixation stability. The findings suggest that optimal fixation can be achieved with a minimum of five screws and a 1.2 mm plate or seven screws with at least a 0.9 mm plate for narrowing applications. For widening applications, six screws with a 0.9 mm plate or eight screws with plate thicknesses ranging from 0.6 mm to 1.2 mm are sufficient to ensure stability. This study highlights the importance of PSI customization in achieving effective stabilization and offers insights for clinicians in improving surgical outcomes. Further research is recommended to explore the impact of dynamic loading, combined movements, and long-term outcomes to refine fixation strategies in T-shaped genioplasty.

## Figures and Tables

**Table 1:** Material properties.

Material	Elastic Modulus (MPa)	Poisson Ratio
Cortical Bone (Mandible)	13700	0.3
Trabecular Bone (Mandible)	1370	0.3
Cortical Bone (Iliac bone Graft)	17000	0.3
Trabecular Bone (Iliac bone Graft)	70	0.2
Titanium Grade V (PSIs and Screws)	110000	0.35

**Table 2:** Quantitative model data.

Models		Total Number of Nodes	Total Number of Elements
Model 1	5-screw hole model - 0.6 mm thickness	1395035	5777441
Model 2	5-screw hole model - 0.9 mm thickness	1407491	5826905
Model 3	5-screw hole model - 1.2 mm thickness	1498098	6214162
Model 4	7-screw hole model - 0.6 mm thickness	1468157	6072027
Model 5	7-screw hole model - 0.9 mm thickness	1493177	6175591
Model 6	7-screw hole model - 1.2 mm thickness	1605234	6248214
Model 7	6-screw hole model - 0.6 mm thickness	1644571	6794383
Model 8	6-screw hole model - 0.9 mm thickness	1679003	6942846
Model 9	6-screw hole model - 1.2 mm thickness	1704459	7063632
Model 10	8-screw hole model - 0.6 mm thickness	1658742	6899637
Model 11	8-screw hole model - 0.9 mm thickness	1914077	8848753
Model 12	8-screw hole model - 1.2 mm thickness	2021808	9170185

**Table 3:** The von Mises stress values on PSIs and screws, and bone displacement amounts at the osteotomy line between bone fragments.

Screws			0.6 mm thickness	0.9 mm thickness	1.2 mm thickness
Narrowing	5 screws	PSI (MPa)	1718.065	1143.125	474.905
		Screw 1-1 (MPa)	472.606	464.112	448.108
		Screw 1-2 (MPa)	420.571	398.047	320.377
		Screw 2-1 (MPa)	632.953	617.176	590.105
		Bone displacement range (mm)	0.36-3.28	0.2-1.8	0.69-1.03
	7 screws	PSI (MPa)	1517.511	805.946	420.219
		Screw 1-1 (MPa)	376.082	339.868	303.043
		Screw 1-2 (MPa)	310.721	287.492	275.687
		Screw 2-1 (MPa)	120.202	82.553	75.097
		Screw 2-2 (MPa)	124.843	121.545	107.199
		Bone displacement range (mm)	0.41-1.67	0.28-1.00	0.42-0.92
Widening	6 screws	PSI (MPa)	999.626	490.248	333.303
		Screw 1-1 (MPa)	393.229	376.926	353.095
		Screw 1-2 (MPa)	248.968	227.901	165.685
		Screw 2-1 (MPa)	249.777	245.787	201.135
		Screw 2-2 (MPa)	203.101	195.897	117.642
		Bone displacement range (mm)	0.34-1.38	0.48-0.97	0.43-0.86
	8 screws	PSI (MPa)	824.526	437.387	241.779
		Screw 1-1 (MPa)	382.844	347.938	334.405
		Screw 1-2 (MPa)	235.847	176.289	119.185
		Screw 2-1 (MPa)	119.191	115.661	111.985
		Screw 2-2 (MPa)	171.333	164.816	140.286
		Screw 2-3 (MPa)	124.013	118.748	113.336
		Bone displacement range (mm)	0.30-0.91	0.44-0.88	0.52-0.79
